# 3D Printing of Strong and Room-Temperature Reprocessable Silicone Vitrimers

**DOI:** 10.1021/acsami.4c16860

**Published:** 2024-12-04

**Authors:** Stefano Menasce, Rafael Libanori, Fergal Coulter, André R. Studart

**Affiliations:** Complex Materials, Department of Materials, ETH Zürich, 8093 Zürich, Switzerland

**Keywords:** elastomers, polymers, dynamic networks, additive manufacturing, recycling

## Abstract

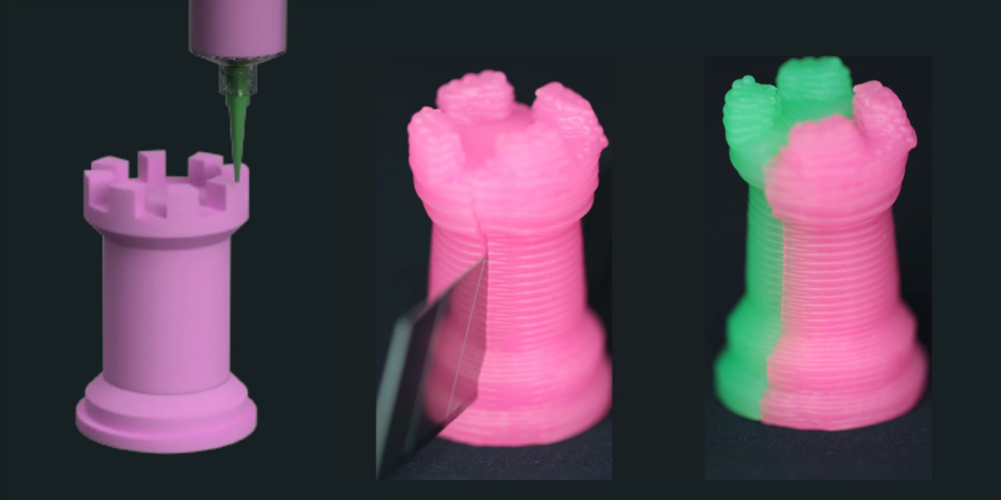

Silicones find use in a myriad of applications from sealants and adhesives to cooking utensils and medical implants. However, state-of-the-art silicone parts fall short in terms of shape complexity and reprocessability. Advances in three-dimensional printing and the discovery of vitrimers have recently opened opportunities for shaping and recycling of silicone objects. Here, we report the 3D printing via direct ink writing of silicone vitrimers into complex-shaped parts with high strength and room-temperature reprocessability. The reprocessing properties of the printed objects result from the adaptive nature of the silicone vitrimer, which can deform under stress without losing its network connectivity. Rheological and mechanical experiments reveal that printable inks can be tuned to generate strong parts with high creep resistance and room-temperature reprocessability, two properties that are usually challenging to reconcile in vitrimers. By combining printability, high strength, and room-temperature reprocessability, the reported silicone vitrimers represent an attractive sustainable alternative to currently available elastomers in a broad range of established and prospective applications.

## Introduction

Silicone vitrimers constitute a unique class of polymers that can combine the high extensibility of elastomers with the self-healing and reprocessability properties of covalent adaptive networks.^1−7^ These combined features arise from highly mobile poly(siloxane) networks that contain reversible dynamic covalent bonds in their molecular structure. Because the glass transition temperature of poly(siloxanes) lies well below room temperature, silicones display the characteristic high extensibility of polymers in the rubbery state.^3,4^ Dynamic covalent bonds that rely on associative interactions allows the covalent network to adapt its topology while maintaining a constant density of cross-links.^8^ Several types of dynamic covalent bonds have been exploited to synthesize silicone vitrimers. Examples include silicones containing dynamic bonds that undergo boronic ester exchange,^6,9^ transamination of vinylogous urethanes,^4^ and transthioesterification of Meldrum’s acids.^3^ However, the self-healing and reprocessability enabled by such dynamic bonds are typically achieved at the cost of the mechanical strength and creep resistance of the vitrimer.

The trade-off between the mechanical strength and the self-healing ability of vitrimers has motivated research on strategies to optimize such antagonistic properties. This effort has led to the development of vitrimers combining multiple types of cross-linkers,^10−14^ reinforced with particle fillers^15−17^ or featuring novel architectures at the meso- or macro-scales.^18,19^ The combination of multiple dynamic bonds^2^ or the use of metal complexes^13,14^ was shown to increase the strength and creep resistance of vitrimers while keeping its self-healing performance. Particle fillers are also effective in reinforcing vitrimers without compromising the adaptive capabilities of the polymer network.^15,16^ The introduction of reinforcing phases in the form of phase-separated domains^19^ or composite laminate architectures^18^ is another promising strategy to create strong and stiff vitrimers with limited creep deformation. Understanding and quantifying the creep behavior of such vitrimers is a crucial next step toward a broader use of these promising materials in real applications.

The ability to shape vitrimers into application-relevant geometries is another important requirement to facilitate the integration of such materials into devices and functional parts. Although vitrimers can be processed through some of the manufacturing technologies available for thermoplastics, research efforts have also been dedicated to the development of additive manufacturing platforms for the fabrication of customized, complex parts that are not accessible using conventional methods.^20−22^ In the context of silicones, various light-based and extrusion-based techniques have been exploited for the additive manufacturing of complex-shaped objects. Silicone-based gels with tunable rheological properties have been formulated to fabricate strong and resilient elastomers via the Direct Ink Writing method.^23,24^ Stereolithography and Digital Light Processing have also been applied to 3D print silicone parts with bespoke intricate geometries.^25,26^ Despite these advancements, reports on additive manufacturing of silicone vitrimers remain rather scarce,^27,28^ with the majority of studies being limited to silicone thermosets with permanent bonds or to vitrimers that are not based on silicone.

Here, we develop and study particle-filled silicone inks for the Direct Ink Writing of silicone vitrimers that combine high strength, reprocessability at room temperature, and predictable creep behavior. The inks contain primarily a silicone prepolymer, a thiol-based dioxaborolane monomer, and silica particles as fillers. The dioxaborolane monomer is utilized to implement dynamic bonds in the vitrimer network, whereas the silica particles are used both for reducing creep and tuning the mechanical properties of the ink and of the final silicone elastomer. First, we investigate the effect of the particle concentration on the rheological properties of the silicone inks. Silicone vitrimers with varying silica contents are then systematically evaluated in terms of mechanical strength and stiffness. An optimal ink formulation is developed to DIW-print complex silicone parts and to assess the material’s self-healing behavior, reprocessability, creep, and elastic recovery compared to previously reported printable vitrimers.

## Experimental Section

### Materials

Vinyl-terminated polydimethylsiloxane (DMS-V22, 200 cSt) and (7.0–8.0% vinylmethylsiloxane)-dimethylsiloxane copolymer, trimethylsiloxy terminated (VDT-731, 800–1200 cSt) were purchased from Gelest, Inc. (USA). Thioglycerol (≥97%), tetrahydrofuran (THF, ≥ 99.9%), toluene (≥99.7%), chloroform-d (99.8%) and molecular sieves (zeolite, 4 Å) were acquired from Sigma-Aldrich (USA). 1,4-phenylenediboronic acid (98%) was obtained from Combi-Blocks, Inc. (USA). 2,2-Dimethoxy-2-phenylacetophenone (DMPA, 99%) was purchased from Acros Organics (USA). SilcPig silicone color pigments were acquired from Smooth-On Inc. (USA), and fumed silica (Aerosil R8200) was purchased from Evonik AG (Germany). The supplier modifies the fumed silica particles with hexamethyldisilazane to impart surface hydrophobicity to this inorganic filler.^29^

### Synthesis Methods

#### Synthesis of Monomer Containing Dioxaborolane Functional Groups (1)

The synthesis of the monomer (1) was accomplished via a condensation reaction involving 1,4-phenylenediboronic acid and thioglycerol. To a 2000 mL, 2-neck round-bottom flask, 16.00 g (96.48 mmol, 2 equiv) of 1,4-phenylenediboronic acid were added, followed by the slow addition of 1440 mL of toluene with stirring until complete homogeneization. The resulting milky dispersion was then connected to a Soxhlet extractor containing molecular sieves and slowly heated to 60 °C. Subsequently, 20.88 g (193.04 mmol, 4 equiv) of thioglycerol were added dropwise, and the temperature was raised to 110 °C. Within minutes, the solution turned clear and was refluxed with vigorous magnetic stirring for 18 h. The resulting reaction solution was filtered, which after solvent removal resulted in the final product as a coarse white powder (28.96 g, yield ∼82%). The 1H NMR spectrum (300 MHz, CDCl^3^) exhibited characteristic peaks at δ 7.82 ppm (s, 4H), 4.74 (m, 2H), 4.49 (m, 2H), 4.17 (m, 2H), 2.81 (m, 4H), and 1.50 (dd, *J* = 11, and 7.4 Hz, 2H).

#### Synthesis of Silicone Vitrimer Prepolymer Mixture (2)

In a 250 mL single-neck round-bottom flask, a mixture of 6.5 g (21.0 mmol, 3 equiv) of monomer (1) and 120 mg (0.46 mmol) of DMPA in 150 mL of THF was prepared. The solids were slowly dissolved in the solvent under stirring. Next, 66.3 g (7.0 mmol, 1 equiv) of vinyl-terminated polydimethylsiloxane were added to the flask while stirring. The solution was subjected to three cycles of freeze–pump–thaw degassing using a Schlenk line to remove all the dissolved oxygen. The minimum pressure reached at the end of each cycle was 10^–6^ bar. The flask was then sealed and placed in an airtight UV curing chamber under nitrogen atmosphere. The solution was kept under magnetic stirring and irradiated for 10 min using a DELOLUX 20 LED lamp (DELO, Germany) that emitted two different wavelengths (400 and 365 nm). The flask was positioned 25 mm away from the light source, and the lamp intensity was set at 30% of its maximum value. The measured intensities at flask distance were 550 mW/cm^2^ and 300 mW/cm^2^ for 400 and 365 nm, respectively. Finally, the solution was concentrated by rotary evaporation to obtain a viscous milky mixture.

### Ink Formulations for Direct Ink Writing

#### Silicone Vitrimer

In a 300 mL Thinky mixer cup (Thinky USA), 69.23 g of silicone vitrimer prepolymer were combined with 46.15 g of trimethylsiloxy-terminated (7.0–8.0% vinylmethylsiloxane)-dimethylsiloxane copolymer, along with 225 mg of DMPA (0.15 wt %) and 300 mg of SilcPig (color: electric pink) at a concentration of 0.2 wt %. The mass ratio of 2:3 between the silicone vitrimer prepolymer and the vinyl-siloxane copolymer was determined empirically to result in a silicone vitrimer with enhanced mechanical properties. After manual mixing, different amounts of Aerosil R8200 silica nanoparticles were slowly added to the resin formulation: 11.53, 23.07, 34.61, and 46.15 g for 10, 20, 30, and 40 phr (parts per hundred of rubber), respectively. The resulting mixture was homogeneized in a planetary centrifugal mixer (Thinky Mixer ARE-250) for 3 min at 2200 rpm.

### 3D-Printing

To enable direct ink writing of structures, we utilized 55 cm^3^ syringe barrels (ADV855BA model) fitted with syringe pistons (ADV830WW model) and a 0.84 mm nozzle (TT18-rigid model) procured from Adhesive Dispensing Ltd., UK. The syringe barrels were manually filled up to 75% of their full capacity and subsequently connected to a 3D-discovery extrusion-based printer (RegenHU SA, Switzerland) to fabricate 1.8 mm-thick films. The resulting films were used to extract dogbone-shaped specimens for quasi-static, creep, elastic recovery and self-healing tests. During printing, the ink with 30 phr of silica was extruded at a rate of 12 mm/s and an applied pressure of around 3.5 bar.

### Curing

To ensure proper curing, the samples were placed in a custom-made UV chamber and purged with nitrogen for 3 min. After that, samples were exposed to the 400 nm UV lamp at 57 mm distance from the light source for 600 s at a constant intensity of 403 mW/cm^2^. As demonstrated in earlier work, the irradiation intensity and time controls the cross-linking density of the silicone vitrimer network.^30^

### Ink Rheology

To evaluate the rheological properties of the inks, a compact rheometer (Anton Paar MCR 302 rheometer) equipped with a sandblasted parallel plate geometry (PP25) with a 1 mm gap was used at a temperature of 25 °C. The apparent viscosities of the inks were determined under steady-shear conditions by varying the applied shear rate from 10^–2^ to 100 s^–1^, followed by a ramp-down from 100 to 10^–2^ s^–1^. Oscillatory measurements were conducted at a maximum amplitude strain of 1% and a frequency of 10 rad/s, while stress–strain flow tests were performed at a shear strain rate of 0.1 s^–1^. Although these testing conditions do not fully replicate the extensional flow that occurs during extrusion, they serve as a proxy for the shear forces applied to the inks during 3D printing. Overall, the rheological measurements were performed under stress-controlled conditions starting at a minimum stress of 1 Pa, with a 10 s integration time per data point.

### Mechanical Characterization of 3D-Printed Parts

The mechanical tests were carried out using dogbone-shaped samples in accordance with the ISO 37 (type 4) standard at room temperature.

The uniaxial tensile tests were conducted using a Shimadzu AGS-X tester with a 100 N load cell, with the samples stretched at a rate of 80 mm/min until rupture. The elastic modulus (stiffness) of the sample is taken as the tangent modulus at 10% elongation (M10), which was determined as the slope of the stress–strain curve at 10% elongation. The values reported for strength, strain at break, elastic modulus and creep are the averages of at least three samples.

Creep tests were conducted in a uniaxial tensile tester (TA.XTPlus, Stable Micro Systems) using a 5 N load cell. The creep behavior of the samples was evaluated by subjecting them to an initial stretch at a rate of 80 mm/min, followed by a static load application of 5, 50, 125, or 200 kPa for a duration of 90 min. After load release, elastic recovery was evaluated by measuring the sample length at regular time intervals.

Stress-relaxation tests were conducted using a uniaxial tensile tester (Instron material testing machine, dual column table top model #5864) using a 100N load cell and equipped with a digitally controlled furnace. The tests were carried out at room temperature (22 °C), 50 and 100 °C. The stress relaxation behavior of the samples was evaluated by subjecting them to an initial stretch at a rate of 80 mm/min until 100% strain was reached. After that the clamps position was fixed and stress relaxation was measured over time for 10 min.

Except for the stress relaxation measurements, all tests were conducted at room temperature.

### Reprocessability Experiment

To assess the reprocessability of the printed silicone, approximately 4 g of the material was chopped into smaller pieces and placed into a mold measuring approximately 40 × 50 mm^2^. A static load of about 0.083 MPa (17 kg) was applied to the material for a period ranging from 16 to 144 h.

### Nuclear Magnetic Resonance

^1^H NMR spectra were recorded on a Bruker AV 300 MHz (Billerica, MA, USA) spectrometer in CDCl_3_.

## Results and Discussion

**Figure 1:**
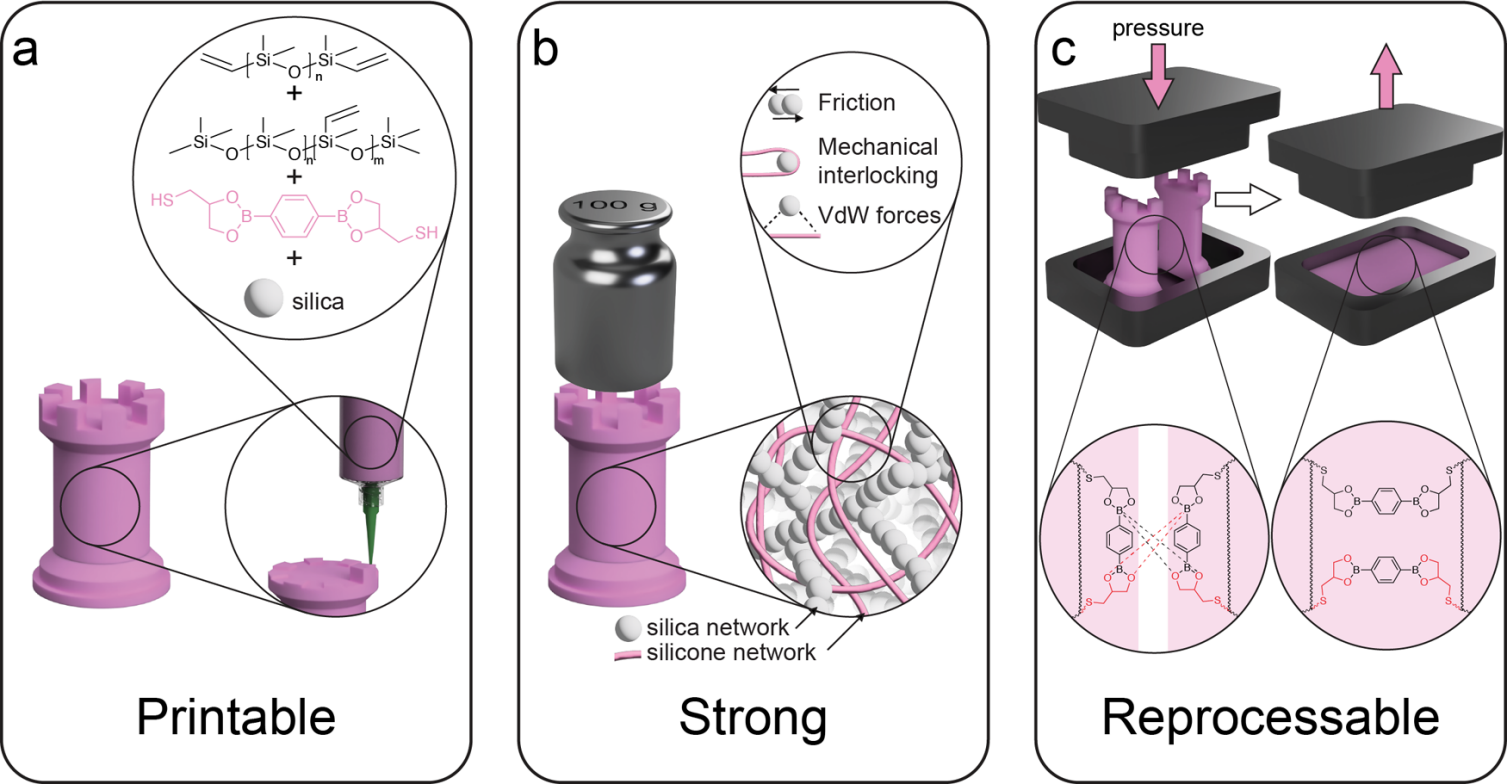
Design of silicone vitrimers for printability, mechanical strength, and recyclability. (a) Network of silica nanofillers is used to tune the rheological properties of a photocurable silicone mixture. (b) Attractive interactions between the silicone-based network and the silica nanofillers are exploited to achieve high mechanical strength. (c) Dioxaborolane groups are introduced in the silicone prepolymer to create dynamic covalent bonds in the cross-linked network.

Silicone vitrimers were made printable, strong, and reprocessable by designing a composite elastomer with distinct molecular and colloidal features at multiple length scales (Figure 1). To achieve printability, it is necessary to formulate inks with rheological properties that satisfy the requirements for 3D printing via Direct Ink Writing. For this purpose, we exploited the network-forming abilities of surface-hydrophobized silica nanofillers suspended in a photocurable silicone mixture (Figure 1a).^31^ In addition to its role as rheology modifier, the silica nanofillers are also used to enhance the mechanical strength of the printed silicone vitrimer after the photocuring step. Such particle reinforcing effect is expected to arise from optimal attractive interactions between the silica nanofillers and the silicone elastomer,^29^ as well as toughening mechanisms enabled by the nanoparticles (Figure 1b).^32,33^ Previous work has shown that the small aggregate morphology and hydrophobic nature of the silica nanoparticles used in this study lead to filler–polymer interactions and optimal chain entanglement that suppress embrittlement and enables effective reinforcement of silicones (Figure 1b).^29^ Finally, recyclability requires a silicone-based molecular network with dynamic covalent bonds. To this end, we designed a silicone prepolymer that contains reactive thiol groups for network formation, as well as dioxaborolane units for the implementation of reversible covalent bonds (Figure 1c).

**Figure 2:**
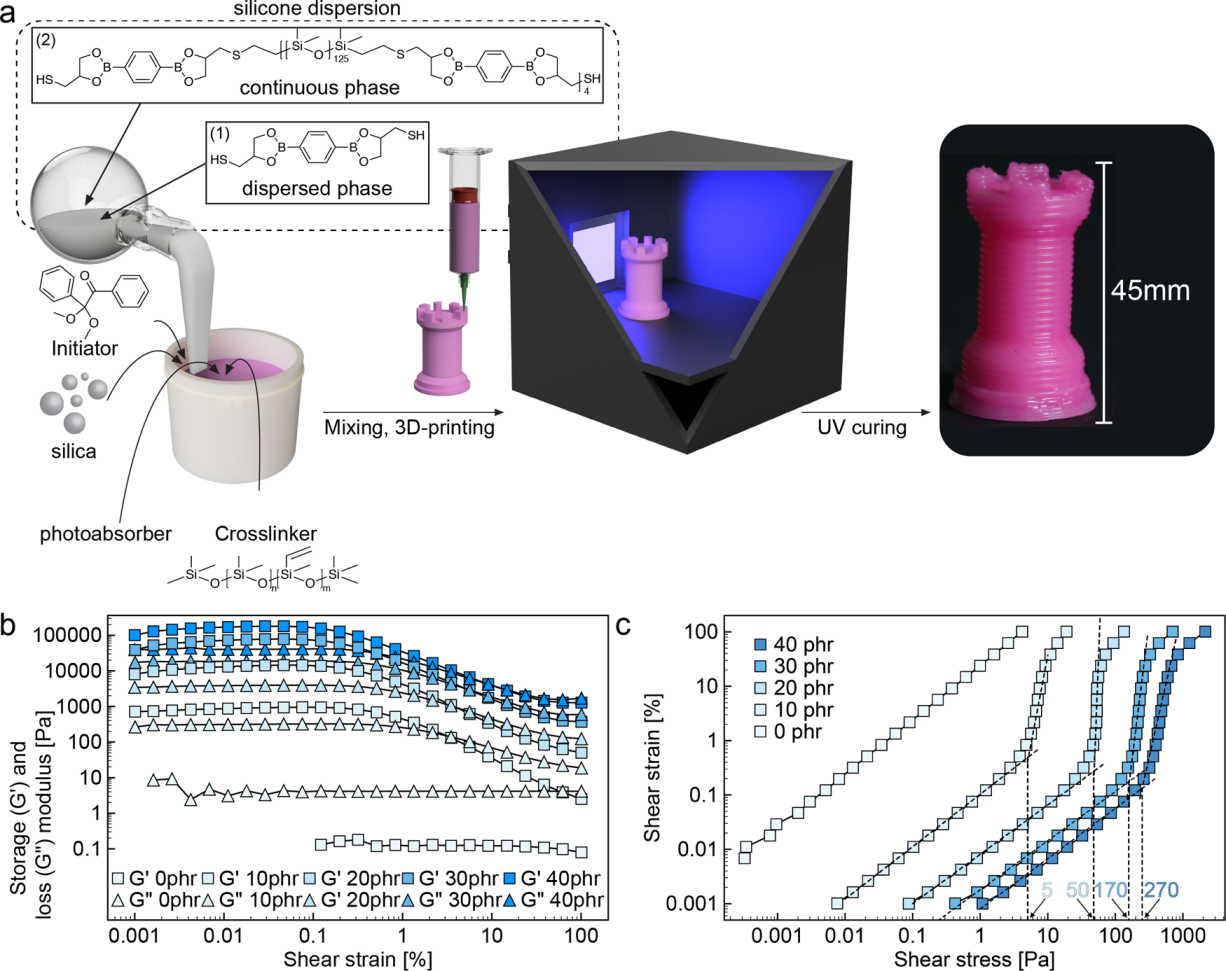
Formulation, rheology, and 3D printing of silica-laden silicone inks. (a) Schematics displaying the mixing, 3D printing, and UV curing of inks containing silicone prepolymer, thiol-ended dioxaborolane, siloxane cross-linker, silica nanofillers, photoinitiator, and photoabsorber. (b) Oscillatory and (c) steady-state rheology of silicone inks containing distinct concentrations of silica nanofillers.

The synthesis of the designed elastomer composite was performed in two main steps. First, a silicone prepolymer containing dynamic groups was prepared through a chain-extension reaction between thiol-functionalized dioxaborolane molecules and vinyl-terminated polysiloxanes. Following our earlier work, an excess of thiols was used in this reaction to ensure the formation of a silicone prepolymer with thiol end groups. Next, the prepolymer was mixed with up to 23.1 wt % (30 parts per hundred rubber, phr) of silica nanofillers, a vinyl-modified silicone cross-linker, a photoabsorber and a photoinititiator to generate a light-curable printable ink (Figure 2a). Because of the low miscibility of thiolated dioxaborolane in silicone, the mixture contained phase-separated domains that rendered the ink optically opaque. Despite the phase separation, the stability of the ink is sufficient to ensure a shelf life of at least 3 months.

Inks with rheological properties suitable for DIW printing were selected based on a thorough rheological characterization of formulations with varying concentrations of silica nanofiller. Rheological measurements were conducted under oscillatory and steady-state shear to quantify the storage modulus (*G*′) and the yield stress (τ_y_), respectively, of the silicone mixtures. A minimum storage modulus in the order of 10^4^ kPa is usually required to enable DIW printing of grid-like structures that resist gravity-driven sagging of filaments. In terms of yield stress, values above 100 Pa are typically needed to prevent capillary-induced distortion of structures printed by DIW.

The rheological measurements revealed that adding silica nanofillers has a major impact on the mechanical properties of the silicone mixtures. In the absence of silica, the mixture shows a clear Newtonian behavior with constant viscosity for shear rates above 0.1 s^–1^ (Figure S1). This viscous response transitions to a viscoelastoplastic behavior upon the incorporation of silica particles at concentrations equal to or above 9.1 wt % (10 phr). The elastic nature of the silica-laden mixtures is evidenced by the fact that the storage modulus (*G*′) is higher than the loss modulus (*G*″) of the material at low applied strain (Figure 2b). Fluidization occurs at the yield stress of the mixture, which is noticeable by a kink in the flow curves obtained under steady-shear conditions (Figure 2c).

The *G*′ value of the silicone mixture was found to increase from 700 to 10^5^ Pa when the silica content changes from 9.1 to 28.6 wt % (10 to 40 phr). Such an increase in silica concentration also enhances the τ_y_ value of the mixture from 5 to 270 Pa (Figure 2c) and changes the ink flow behavior from Newtonian to strongly shear-thinning (Figure S1). Our experiments indicate that a minimum silica content of 23.1 wt % (30 phr) is needed to reach the rheological response suitable for DIW printing. Direct Ink Writing of inks containing 23.1 wt % of silica enabled printing of distortion-free silicone structures, confirming the adequate rheological behavior of this formulation (Figure 2). UV curing of such structures with an LED at light intensity of 400 W/cm^2^ for 10 min allowed for the fabrication of strong and resilient elastomers with complex geometries (Figure 2a).

**Figure 3:**
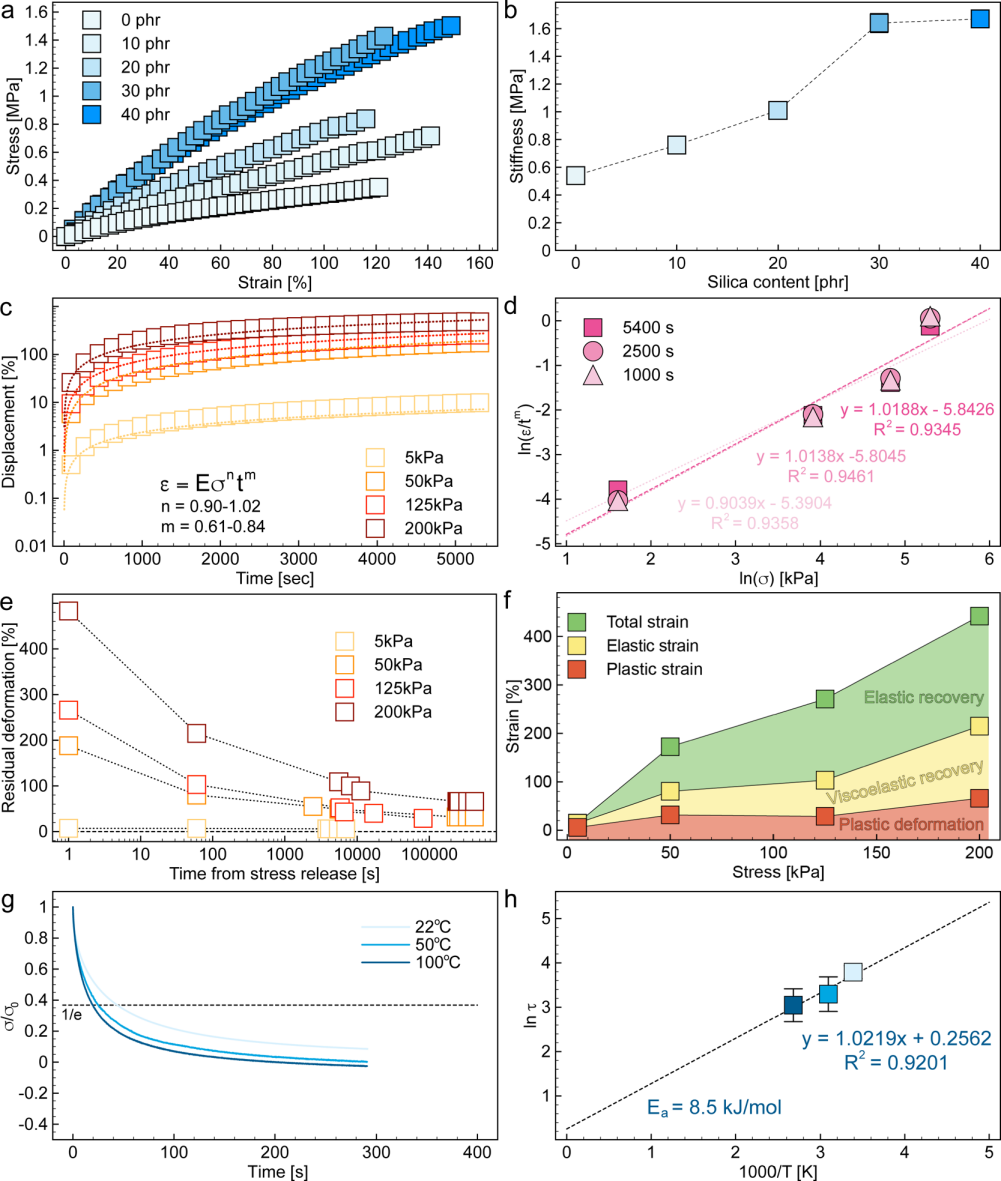
Mechanical properties of printable silicone vitrimers. (a) Stress–strain response under tensile mode for silicones without and with 9.1–28.6 wt % of silica nanofillers, which corresponds to 0 to 40 phr. (b) Stiffness of silicone vitrimers as a function of silica content. (c) Creep displacement as a function of time for silicone vitrimer samples containing 23.1 wt % silica subjected to distinct stress levels. (d) Double logarithmic plot used to extract the stress exponent, *n*, for samples tested under different stress levels. (e) Evolution of the residual strain of the samples after removal of the stress applied during the creep test. (f) Effect of the applied stress on the total strain, the elastic strain, and the plastic strain obtained from the elastic recovery experiment. The plot highlights the fraction of the total strain that is recovered elastically (<1 min) and viscoelastically (1 min–1 day), as well as the irreversible plastic deformation. (g) Stress relaxation curve obtained for the optimal silicone vitrimer (23.1 wt % silica) at three different temperatures. (h) Arrhenius plot depicting the effect of temperature on the relaxation time scale of the silicone vitrimer network.

To quantify the mechanical properties of the printed silicones after curing, we carried out tensile tests on samples with silica concentrations varying from 0 to 28.6 wt % (40 phr) (Figure 3a,b). The tensile tests were performed on dogbone specimens at a strain rate of 80 mm/min using a universal mechanical testing machine. The stress–strain response of a reference silicone sample without silica particles confirms the high extensibility of the elastomer. The addition of silica concentrations in the range 9.1–23.1 wt % (10–30 phr) enhanced both the elastic modulus (stiffness) and the rupture strength of elastomer while keeping the strain-at-rupture relatively constant (Figure 3a). This reinforcing effect saturates at a silica content of 23.1 wt % (30 phr). For this concentration, a 3-fold increase of the elastic modulus and 4-fold increase of the rupture strength is observed relative to the silica-free vitrimer. Such a reinforcing effect suggests that silicone network interacts strongly with the silica particles. This was also found to be the case for recently reported epoxy-based vitrimers containing filler particles.^15,16^

Creep analysis was carried out in silicone vitrimer samples containing 23.1 wt % (30 phr) by measuring the relative displacement of the dogbone samples while subjected to a constant stress in the range 5–200 kPa. The obtained data show the typical pattern expected for primary creep, in which the strain first increases sharply with time before reaching a relatively constant rate as the material approaches the secondary creep regime (Figure S2). The primary creep data can be described using the Miller-Norton power law,^34,35^ which predicts the following scaling relation for the creep strain (ε): ε ∼ σ^*n*^*t*^*m*^, where σ is the applied stress and *t* is the elapsed time. Such a dependence has already been observed in previous work on other vitrimers.^36^ Fitting the creep scaling relation to our experimental data leads to *m* values in the range 0.61–0.84 (Figure 3c). The estimated time exponent, *m*, is in good agreement with values previously measured for an epoxy vitrimer above the glass transition temperature (*m* = 0.7).^15^

In terms of physical mechanisms, creep deformation in vitrimers has been attributed to the elastic stretching of the polymer chains between cross-linkers before a percolating network of loaded cross-links is fully developed.^15^ To gain further insights into the mechanism controlling the creep strain of the silicone vitrimer, we used our experimental data and the values of *m* to also estimate the stress exponent, *n*. This was accomplished by displaying the normalized creep strain, ε/*t*^*m*^, as a function of the applied stress in a double logarithmic plot (Figure 3d). Fitting of the data led to stress exponent values, *n*, in the range 0.90–1.02. Such an analysis indicates a linear dependence between the creep strain and the applied stress, a correlation expected for linear elastic materials. This result suggests that the creep deformation of the vitrimer is dominated by the elastic properties of the vitrimer network.

The strong elastic nature of the silicone vitrimer allows the dynamically cross-linked network to recover most of the creep deformation after the applied stress is released. To illustrate this recovery, we measured the changes in length of the specimens as a function of time right after the creep experiment. The length was converted into residual strain to compare the elastic recovery of samples subjected to distinct stress levels in the creep test (Figure 3e). The data reveals that a significant fraction of the total creep strain is elastically recovered within the first minute after the release of the applied mechanical load (elastic strain, Figure 3f). This is followed by a slower decrease of the residual strain, which we attribute to the viscoelastic recovery of the material. After approximately 1 day, the absolute strain eventually reaches a constant value that is assigned to the irreversible plastic deformation of the specimen (plastic strain, Figure 3f). As expected, the measured elastic and plastic strains increase with the stress applied in the creep test. Regardless of the applied stress, our experiments reveal that 66–85% of the total creep strain is recovered upon mechanical unloading. Up to 52% of such recovery is purely elastic and occurs almost instantaneously (<1 min). The other 33–61% of the recovery takes place within 24 h and involves the viscoelastic deformation of the elastomer.

To gain insights into the molecular mechanisms controlling the deformation of the mechanically loaded vitrimer network, we performed stress relaxation measurements on samples containing 23.1 wt % silica at three distinct temperatures (Figure 3g). By measuring the effect of temperature on the relaxation time scale of the vitrimer network (τ), it is possible determine the activation energy of the transesterification exchange reaction (*E*_a_) using the Arrhenius’ equation: τ ∼ *e*^*E*_a_/*RT*^, where *R* is the universal gas constant and *T* is the absolute temperature.^37^ The relaxation time scale of the silicone network was experimentally quantified by fitting the obtained stress decay curves with the exponential function: σ/σ_0_ ∼ *e*^–*t*/τ^, where σ is the actual stress, σ_0_ is the initial applied stress and *t* is the elapsed time (Figure 3g). The experimental results show that the relaxation time scale decreases from 45 s to 21 s upon a temperature increase from 22 to 100 °C. By fitting these data with a linear equation in an Arrhenius plot (Figure 3h), we estimate an activation energy of 8.5 kJ/mol for the optimized silicone vitrimer. The *E*_a_ value obtained from this analysis falls within the lower boundary of the typical range 8–77 kJ/mol previously reported for vitrimers containing dynamic bonds based on boronic esters, which is possibly attributed to the low cross-linking density and the intrinsically high chain mobility of Si–O–Si backbone of the network.^9,37,38^ This analysis indicates that the plastic deformation of our cured silicone vitrimer is governed by the exchange reactions of the dioxaborolane groups within the dynamic network and is not limited by the mobility of the siloxane chains.

**Figure 4:**
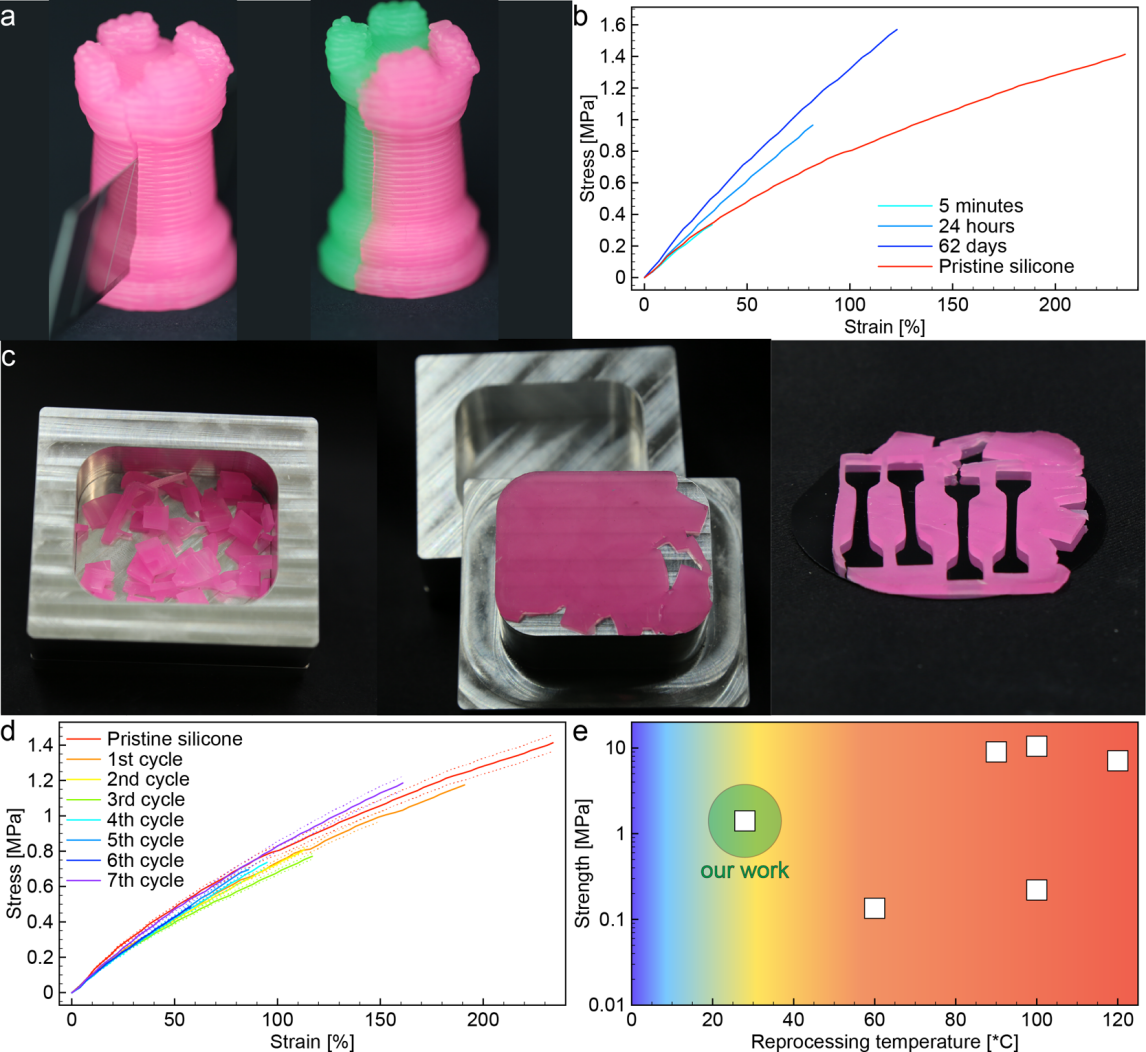
Reprocessability and room-temperature recycling behavior of silicone vitrimers containing 23.1 wt % silica nanofillers. (a) Photographs depicting the amendability of printed silicone vitrimers. (b) Tensile stress–strain response of silicone specimens after amending compared to a pristine sample. (c) Photographs displaying a reprocessing cycle of the silicone vitrimer at room temperature. A fragmented sample is compressed in a mold to generate a silicone monolith. Dogbone specimens are extracted from the monolith for mechanical testing. (d) Tensile stress–strain curves obtained from vitrimer samples subjected to various reprocessing cycles. (e) Rupture strength and reprocessing temperature of the silica-containing silicone vitrimer compared to other printable silicones reported in the literature. The literature values include data obtained for printable silicone networks containing associative exchange groups^27,28^ or supramolecular groups.^41−43^

The low activation energy of the boronic ester exchange reactions imbues the optimized silicone vitrimers with unique self-healing, reprocessability and amendability properties. This complements existing approaches to enable sustainable reprocessing^39^ and shape reprogrammability^40^ of 3D printed polymers. Notably, the high mobility of the silicone chains allows the dynamic network to achieve these adaptive features at room temperature by simple contact or under low applied pressures. To demonstrate the amendability of the silicone vitrimer, we cut a tower-shaped printed part in two pieces and put them back in contact exerting gentle manual pressure. After about 5 min, the two parts were strongly amended and could no longer be pulled apart (Figure 4a). The amendability was quantified by performing tensile tests on specimens that were cut and put back in contact to self-heal for contact times varying from 5 min up to 70 days. The stress–strain curves obtained before and after amending the samples showed that 30, 70, and 100% of the initial strength of the silicone vitrimer could be recovered in 5 min, 16 h and 10 days, respectively (Figures 4b and S3). Remarkably, the strength recovery was accompanied by a stiffening effect that increased the elastic modulus of the specimen by 14% after 62 days. Such a stiffening behavior may result from the presence of excess free dioxaborolanethiols that can further react and strengthen the silicone vitrimer over time.^18^ It is important to note that such an increase in elastic modulus was found to take place predominantly within the first week after preparation of the sample (Figure S4).

To assess the recyclability and reprocessability of the vitrimer, we fragmented the elastomer in multiple small pieces and compressed them in a mold at room temperature under an uniaxial pressure of about 80 kPa for 24 h (Figure 4c). The applied pressure transformed the fragmented pieces into a continuous, homogeneous slab of vitrimer. Dogbone samples were extracted from the compressed slab to assess the level of strength recovery achieved through this process. The reprocessed sample displayed an elastic modulus (*E*) of 0.99 MPa and a rupture strength (σ_r_) of 1.06 (Figure 4d). Such *E* and σ_r_ values correspond to, respectively, 80 and 75% of the stiffness and strength levels obtained with the pristine specimens. The reprocessing cycle was repeated 7 times to evaluate the ability of the material to recover its mechanical properties after multiple recycling rounds. Stress–strain curves measured after each cycle indicate that the stiffness of the vitrimer is not affected by recycling. Conversely, the rupture strength was found to be dependent on possible defects remaining in the sample after compression. Although this led to a stronger variation in the σ_r_ values between cycles, the average strength of recycled vitrimers still reached 65% of that in the original sample. Despite the long reprocessing time used in this study, previous work has shown that 15–40% of the initial strength of the vitrimer network is recovered already in the first 5 min of contact.^30^ Since the reprocessing mechanism relies on the high mobility of the silicone chains and the low activation energy of the exchange reaction of the dioxaborolane groups, increasing the temperature of the mold is expected to increase further the strength recovery ability of the silicone vitrimer. This prediction is based on the higher chain mobility and lower relaxation time observed when the temperature is increased from 22 to 100 °C (Figure 3g,h).

The broader significance of the obtained mechanical and reprocessability results was evaluated by comparing the optimized silicone vitrimer with previously reported printed, reprocessable silicones. This was accomplished by displaying in a single plot the rupture strength and the reprocessing temperature of our elastomer and of silicone-based vitrimers reported in the literature (Figure 4e). The comparative analysis shows that the optimized silicone vitrimer features a unique combination of high rupture strength and low reprocessing temperature, which cannot be achieved by dynamic silicones synthesized in earlier studies. For example, silicone vitrimers printed by selective laser sintering can reach high mechanical strength (7.1 MPa) but need temperatures of at least 120 °C to be reprocessed. Such high reprocessability temperature results from the relatively high activation energy (57–81 kJ/mol)^27^ of the pyrazole urea exchange reactions present in this vitrimer. This comparison highlights the importance of a low activation energy for the reprocessability of the silicone vitrimers containing dioxaborolane functional groups.

With the ability to be reprocessed at room temperature, the strong silicone vitrimer reported here offers a more sustainable alternative to currently available silicone-based elastomers. It is important to note though that further research is needed to reach the unique combination of high strength and high elongation of commercial silicones. While the strength of our silicone vitrimer reaches a level comparable to that of highly optimized commercial silicones cross-linked with permanent bonds, its elongation at break is still lower (up to 220%) compared to available silicone products, such as DragonSkin FX-Pro and Ecoflex 00–20 (763–845%).

**Figure 5:**
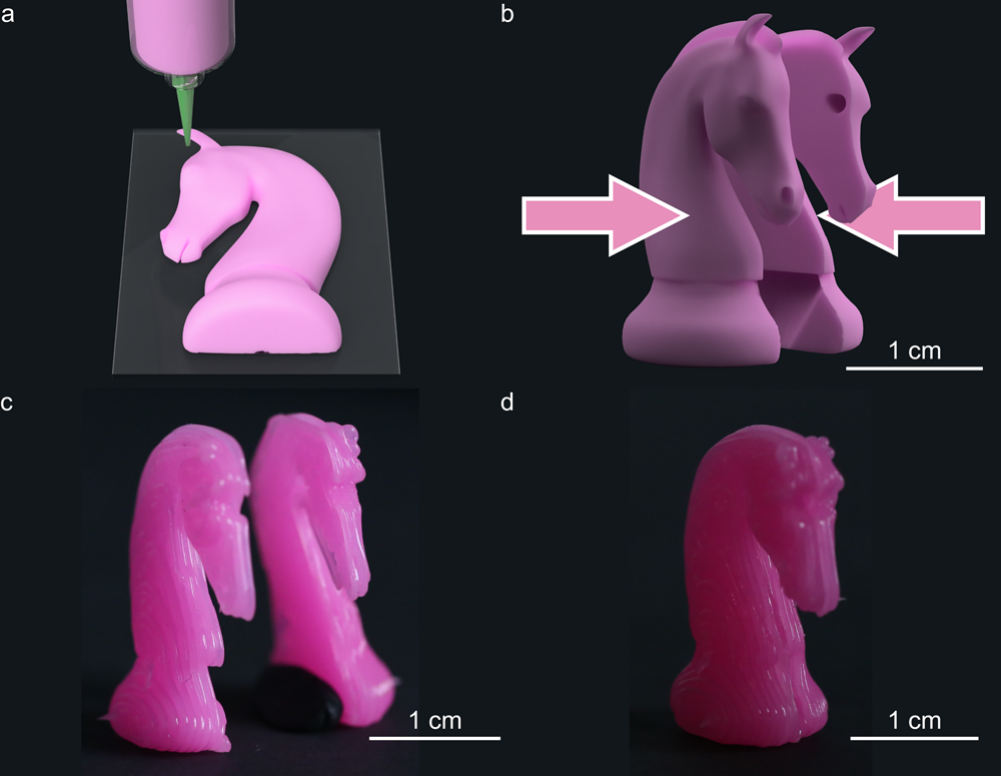
Manufacturing of a complex-shaped silicone part by 3D printing followed by amending. (a,b) Schematics depicting the (a) DIW printing of one-half of a horse chess piece in a horizontal orientation and (b) merger of two printed halves into the full piece by room-temperature amending. (c,d) Experimental realization of the (c) horizontally printed half part (top view) and (d) vertically standing full horse chess piece with overhang.

In addition to the sustainability aspect, the possibility to amend the silicone parts at room temperature opens new routes for the fabrication of complex-shaped silicone structures. For example, tall structures with overhangs can be sliced into parts that are easier to print separately and later amended into more complex three-dimensional objects. We demonstrate this potential by printing a horse chess piece as an example of a vertically tall object with a large overhang (Figure 5). DIW printing of such a geometry in a single run typically requires the cumbersome use and removal of support material. Instead, the amendable nature of the silicone vitrimer greatly facilitates the fabrication of this complex part, which can be achieved by first printing two halves of the horse pieces horizontally (Figure 5a,c) and later amending these printed halves into the full three-dimensional structure (Figure 5b,d). Since the strength of the amended parts can reach the level expected for the pristine material (Figure 4b), this printing strategy provides an efficient means to fabricate complex geometries at potentially higher speeds, lower waste and reduced manufacturing costs (Figure S5).

## Conclusions

Silicone-based inks modified with silica nanofillers can be printed into complex-shaped vitrimer parts displaying high mechanical properties, room-temperature reprocessability and predictable creep behavior. The silica particles are crucial to tune the rheological properties of the ink within the desired printable range and to create a reinforced vitrimer network that is both strong and adaptive. Silicone formulations with 23.1 wt % silica particles were found to be optimum in terms of ink rheology and mechanical properties of the photocured vitrimers. Mechanical tests with this optimized composition revealed that the creep deformation of the silicone vitrimer is well described by a simple empirical model. By fitting this model to our experimental data, we inferred that the creep behavior is dominated by the elastic deformation of the cross-linked vitrimer network. Such physical interpretation is supported by strain recovery tests, which shows that up to 52% of the creep deformation is elastically recovered upon stress release. Compared to other printable dynamic polymers, our silicone networks feature an unusual combination of high mechanical strength with reprocessability at room temperature. These remarkable properties allow printed parts to be amended and reprocessed at 25 °C at mild applied pressures. Notably, the amended and recycled parts exhibit mechanical properties comparable to the pristine material. The printability, high strength, and room-temperature reprocessability of the obtained silicone vitrimers open new opportunities for the sustainable manufacturing and use of elastomers in applications ranging from soft robotics to medical implants and wearable devices.

## Supporting Information Available

Apparent viscosity of silicone-based inks, creep behavior of silicone vitrimers, evolution of the strength and modulus of amended silicone vitrimers, and 3D printed amended structures (PDF)
