# Supplementary material for: 3D Printing of Strong and Room-Temperature Reprocessable Silicone Vitrimers
Source: ACS Appl Mater Interfaces. 2024 Dec 4;16(50):69919–28. doi: 10.1021/acsami.4c16860 (PMC11660043; doi:10.1021/acsami.4c16860)
Supplement: Supplementary file 1 — am4c16860_si_001.pdf [file am4c16860_si_001.pdf]

Supporting Information

3D Printing of Strong and Room-temperature Reprocessable Silicone Vitrimers

Stefano Menasce, Rafael Libanori, * Fergal Coulter, André R. Studart *

Complex Materials, Department of Materials, ETH Zürich, 8093 Zürich, Switzerland

* corresponding authors: rafael.libanori@mat.ethz.ch, andre.studart@mat.ethz.ch

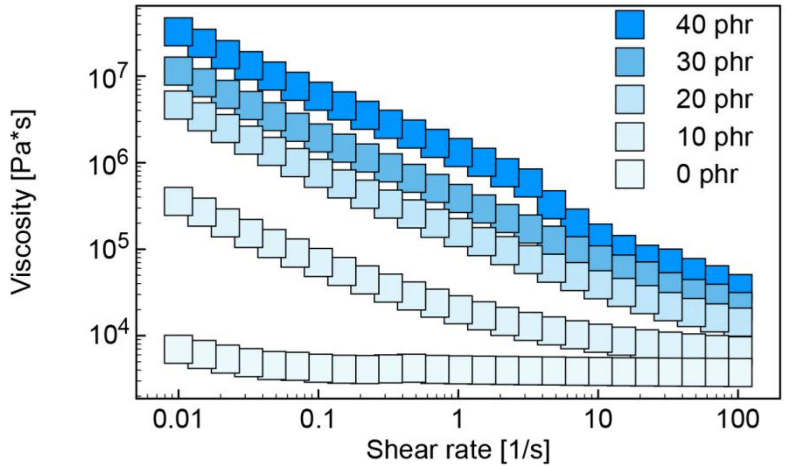

Figure S1. Apparent viscosity as function of shear rate for silicone-based inks containing different concentrations of silica nanoparticles.

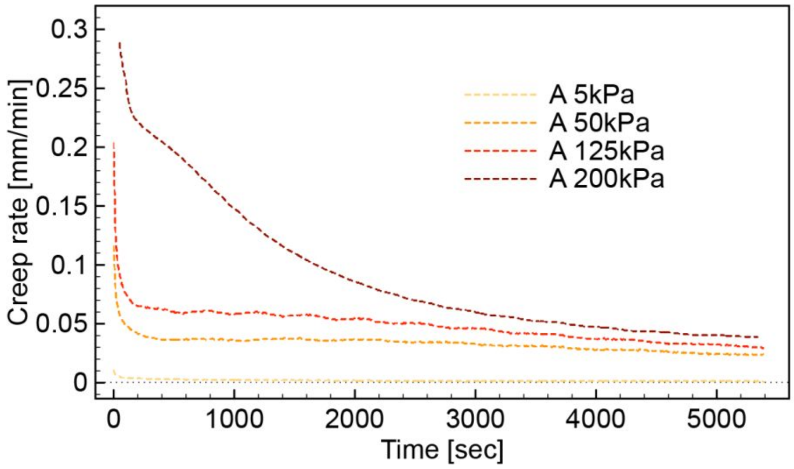

Figure S2. Creep rate as a function of time obtained for the silicone vitrimer sample containing 23.1wt% silica subjected to different applied stresses.

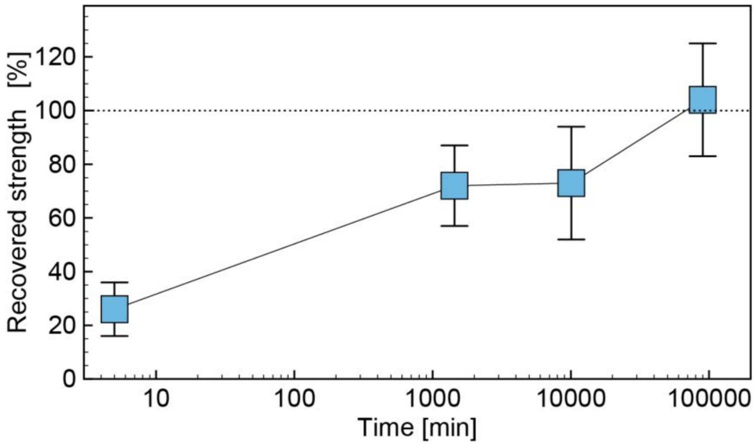

Figure S3. Evolution of the recovered strength for the silicone vitrimer containing 23.1wt% silica nanoparticles. The recovered strength corresponds to the percentual ratio between the strength of the amended sample and the strength of the pristine sample.

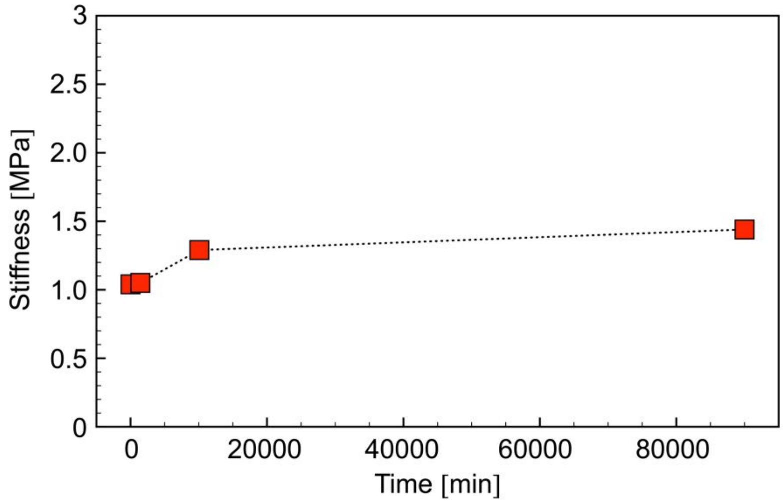

Figure S4. Evolution of the tangent modulus for the silicone vitrimer containing 23.1wt% silica nanoparticles.

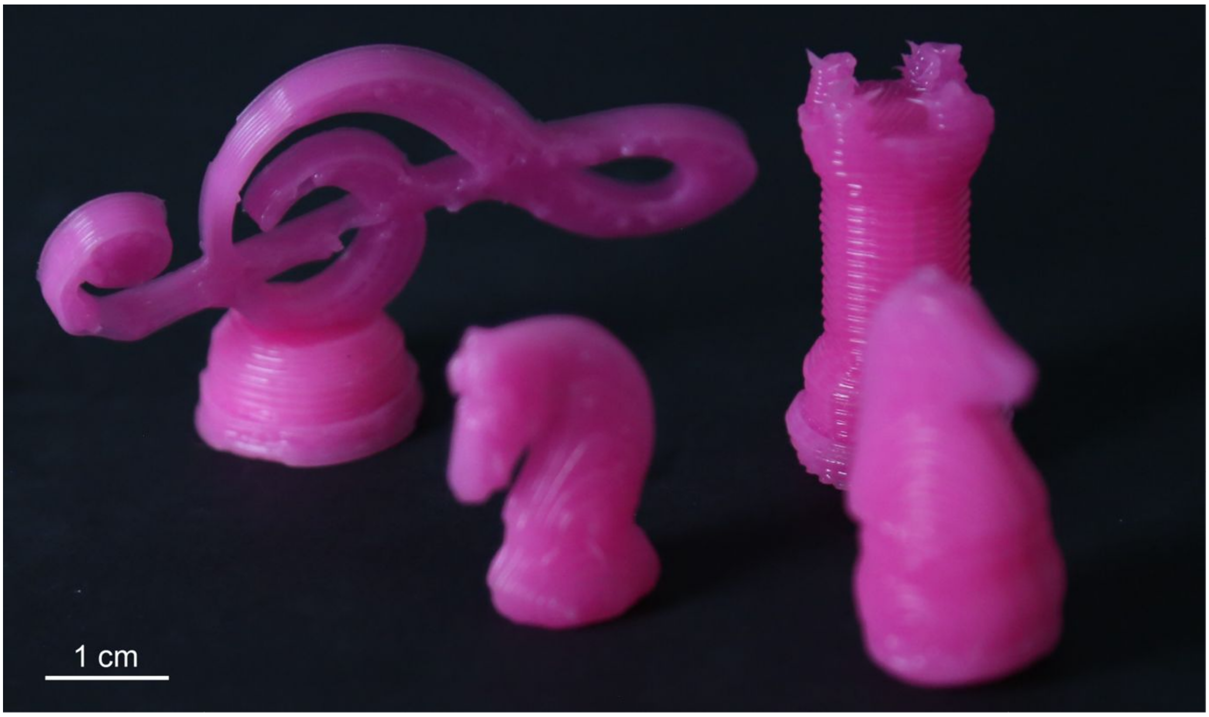

Figure S5. Printed amendable structures manufactured from the optimized silicone vitrimer formulation.
